# Understanding the barriers and enablers to participation in vaccine trials in a pregnant population from diverse ethnic background in an inner-city UK hospital

**DOI:** 10.1371/journal.pone.0312799

**Published:** 2024-10-30

**Authors:** Essra Youssef, Anna Calvert, Vanessa Greening, Dominique Pearce, Suzannah Wright, Emma Eccleston, Lolade Oshodi, Paul Heath, Tushna Vandrevala

**Affiliations:** 1 School of Nursing, Allied and Public Health, Faculty of Health, Science, Social Care and Education, Kingston University London, London, United Kingdom; 2 Centre for Neonatal and Paediatric Infection and Vaccine Institute, St George’s, University of London, London, United Kingdom; 3 Women’s Health Research, University College London Hospitals NHS Foundation Trust, London, United Kingdom; KEMRI-Wellcome Trust Research Programme, KENYA

## Abstract

**Background:**

Vaccination during pregnancy is an important healthcare intervention for safeguarding the health of the mother and their infants. Ethnic disparities in recruitment to vaccine research studies during pregnancy potentially contribute to health inequalities. The aim of the current study was to explore the barriers and enablers influencing the willingness of pregnant women from ethnic minority backgrounds to participate in vaccine research studies.

**Methods and findings:**

Semi-structured qualitative online interviews were conducted with 23 pregnant women from diverse ethnic backgrounds in the UK. Interviews were transcribed verbatim, and thematically analysed. Our findings suggest that participants perceived vaccines and vaccine research, in principle, to be beneficial to the individual and to society, and understood the value of vaccination in mitigating severity of disease and protecting the health of mothers and their infants. Apprehension over the safety of vaccination in pregnancy was common and reduced willingness to participate. For those that decided to participate in vaccine trials in pregnancy, this was seen as an act of solidarity, a way to contribute to a collective responsibility for the public health of the community. Personal and community connections and representation—seeing people from their own communities represented in in the recruitment process shapped decisions about vaccine trial participating. Trust and mistrust in health systems, shaped by past experiences of interacting with healthcare professionals were likely to inform whether they would consider participating. Practical considerations such as excessive time commitments related to study procedures, travel and organising childcare were barrier to participation. The level of invasiveness of trial procedures were also a concern, although increased monitoring during the trial was seen as a potential benefit, mitigating some safety concerns.

**Conclusions:**

Our study reinforcing previously identified barriers to vaccine participation among pregnant women from diverse ethnic communities. This study underlines the need to develop tailored interventions that focus on fostering trust with the aid of community engagement to understand cultural contexts, establishing authentic representation, and address practical considerations, to contribute to enhancing vaccine trial participation in pregnancy in those from diverse ethnic communities.

## Introduction

Vaccination during pregnancy is an important healthcare intervention to improve the health of both the mother and her infant against infections such as tetanus, pertussis (whooping cough), respiratory syncytial virus (RSV), influenza and, more recently COVID-19 [1]. The importance of vaccination in pregnancy is becoming increasingly recognised, with the potential to extend this preventative strategy to other pathogens. A vital part of the success of vaccination programs is the confidence in the pregnant population to engage in public health interventions. Several previous studies have demonstrated that pregnant individuals from diverse ethnic communities are less likely to choose vaccination compared to white pregnant people [2–5]. Although one study found that vaccination rates are higher in Asian women compared with other ethnicities [6], several studies have consistently found that individuals from Black communities are least likely to accept vaccination [5–7].

Despite the United Kingdom having one of the lowest maternal mortality ratios in the world [8], persistent disparities in maternal outcomes persist between different ethnic groups. Maternal mortality rates are nearly four times higher in those of black ethnicities compared to those of white ethnicity [9], with significant disparities also evident among those of Asian and mixed ethnicity [9]. Various factors contribute to these disparities, with an important factor being the differences in healthcare service access, along with specific barriers and enablers that influence engagement with healthcare services.

The COVID-19 pandemic posed an unprecedented global health challenge and brought to the forefront issues of vaccine hesitancy and lower vaccine acceceptance rates in ethnic communities [10]. Vaccine hesitancy is defined as delay in acceptance or refusal of vaccination despite availability of vaccination services [11] and ethnic communities experienced consistent barriers towards vaccine uptake [10, 12]. Recent surveys from the UK during the COVID-19 pandemic, showed the highest rates of vaccine hesitancy were among people of Black ethnicity (71.8%), followed by Pakistani/Bangladeshi (42.4%) and Mixed ethnicity (32.4%) [13]. In contrast, only 15.6% of White British or Irish ethnic groups were vaccine hesitant [13]. A consistent barrier to vaccination uptake in people from ethnic minorities is concern about the safety of vaccines [14] and perceived lack of representation of their communities in vaccine trials [12]. This is compounded by existing poor recruitment and underrepresentation of people from ethnic minorities in clinical trials in the UK, Europe and the USA [15, 16]. The apprehension about the safety and potential side effects of the COVID-19 vaccine, and the decision to make pregnancy an exclusion criteria for the early trails significantly impacted individuals’ decisions to participate in later maternal vaccine programmes. The lack of sufficient safety data about use of the vaccine in pregnancy intensified expectant mothers’ feelings of uncertainty towards the vaccine [17].

Given the safety concerns engendered by vaccine administration in pregnancy, active participation of pregnant people in clinical vaccine research is of paramount importance to generate robust data about vaccine safety and efficacy specific to this group. Pregnancy is a common excluding criterion for intervention research, often, because of a perceptions of greater susceptibility to harm, and in some cases this group are classified as a “vulnerable population” [18]. Engaging pregnant individuals from diverse ethnic backgrounds is instrumental in ensuring that approved vaccines are safe and effective across all ethnicities. This inclusive approach holds the potential to alleviate the observed disparities in maternal care outcomes in the United Kingdom.

This paper explores the barriers and facilitators influencing the willingness in a pregnant population from ethnic diverse backgrounds to participate in vaccine research studies.

## Methods

### Study design

The study utilised a qualitative research approach with semi-structured interviews of pregnant people from ethnic diverse backgrounds. Data analysis was conducted using thematic analysis, as described by Braun and Clarke [19, 20].

### Study setting

The study was conducted in a single centre, at a large teaching hospital in South West London. The hospital serves a diverse population of 1.3 million people, a large proportion of which are from Black, Asian and Minority Ethnic (BAME) backgrounds. According to the UK Office for National Statistics, 35.4% of South West London residents are from BAME backgrounds [21].

## Participants and recruitment

A purposive sampling strategy [22], was used between November 2022 and September 2023 to recruit ethnically diverse participants, primarily those from Black and South-Asian backgrounds. The sample size was guided by the concept of data saturation [23], which is when further data collection is unlikely to yield new information or insights related to the research question.

Participants were recruited in two ways: (1) In-person by a research midwife when attending the hospital after the first routine scan appointment at either week 12 or week 20, and (2) through mail outs in which an invitation letter was sent to women who had attended a 12-week scan appointment. The study was also advertised through promotional posters displayed in the antenatal care clinical areas and online media platforms. Participants were included in the study if they were eligible for participation in any pregnancy vaccine trial, from an ethnic minority group, willing to give informed consent for the study and aged 16 years or over. Once identified, participants were given a participant information sheet and had the opportunity to discuss the study with a member of the research team before signing an informed consent form. Participants either signed the consent form physically in the presence of the research midwife, or at a later date remotely with an electronic signature.

Out of the initial outreach to 1,041 individuals through postal contact and 281 individuals through in-person engagement, a total of 31 pregnant women provided informed consent for inclusion in the study. During the course of the study, 8 participants were inadvertently lost to follow-up, resulting in a final interview cohort of 23 pregnant women.

### Data collection

Interviews were conducted between November 2022 and September 2023. The interview schedule included approximately ten open-ended questions that focused on participants’ attitudes to vaccines and perceptions of barriers and enablers to vaccine research participation during pregnancy. Some examples questions are: *“Tell me more about why you would or/choose not to participate in vaccine research studies*?*”*, *“Is participating in vaccine research different when you are pregnant*? *Why*? *Why not*?*”* (the full topic guide is available in S2 File). Interviews were conducted by a female, paediatric medical doctor [redacted], experienced in qualitative research. The interviewer had no relationship with the interviewees prior to conducting the interview. Interviews were conducted remotely in English using Microsoft Teams (which has built-in privacy features including encryption), and lasted approximately 20–30 minutes.

The interviewer reflected on each of the interviews, considering personal observations, expectations and biases related to the research. She particularly considered how her position as a white female doctor who had herself recently had a baby might affect her views on vaccine trial participation and the way in which she framed questions around this. More importantly she reflected on the way that these characteristics might be perceived by participants. Before each interview, and building on previous interviews, she considered how she could create a safe forum in which views could be shared honestly and openly. The interviewer kept a reflexive diary [24] to record personal observations, expectations and biases related to the research. Memos were also created during and after each interview to mitigate bias and provide an audit trail.

### Data analysis

The interviews were recorded, encrypted, and automatically transcribed using Microsoft Teams live transcription feature. The transcriptions were checked by a member of the research team, who amended them for accuracy and removed personal information to maintain participants anonymity. Transcripts were then imported into NVIVO QSR International Version 12 for qualitative analysis. The data was explored using both inductive and deductive thematic analysis [19, 20].

The initial stage of the analysis was performed by [redacted] who familiarised themselves with the entire data set before proceeding with coding. [Redacted] then generated codes using an inductive approach, whilst ensuring that all data received equal attention. Afterwards, deductive coding was performed on the data (in consultation with author [redacted]) to identify barriers and enablers to vaccine research during pregnancy and how participants positioned themselves with relation to their diverse ethnic backgrounds. Codes were refined and consolidated through consultations and discussions between team members [redacted]. This iterative process aimed to ensure the comprehensive exploration of relevant themes and their connections within the data.

## Ethical consideration

NHS Research ethical approval was granted by the Wales Research Ethics Committee 3 (REC ref 22/WA/0237).

## Findings

### Participant characteristics

A total of 23 participants took part in the study, representing diverse ethnic backgrounds. 8 (34.8%) participants were of South Asian ethnicity, 7 (30.4%) were of Black ethnicity (5 Caribbean and 2 African), 4 (17.4%) were of mixed ethnicity (2 White-Asian and 2 White-Black, 2 (8.7%) were South American and 2 (8.7%) were of other Asian ethnicities. The median age among participants was 33 years (with a standard deviation of 4 years), and the majority, 19 (82.6%), had one or more children at the time of the interview.

The main findings will be discussed under four main themes identified from the data: General perceptions of vaccines and vaccine research, Identity and belonging, (Mis)trust in healthcare and Practical considerations.

### General perceptions of vaccines and vaccine research

Our participants from diverse ethnic communities expressed attitudes towards vaccine and vaccine research which aligned with those seen from individuals from a range of backgrounds [25, 26]. This included the belief that vaccines and vaccine research are beneficial to the individual and society, an altruistic drive that motivates them to participate, and performing a balancing act between personal risks and benefits.

Across minority ethnic backgrounds, those interviewed in our study perceived vaccines as an important healthcare intervention for both adults and children. Participants demonstrated a good degree of knowledge and understanding of the role vaccines play in mitigating the severity of disease and protecting the health of both the mothers and their infants. Additionally, they acknowledged the necessity for clinical trials to provide robust data confirming the safety and effectiveness of vaccines and the importance of clinical trials to include a diverse population.

"*I do think it’s important. I mean, if people didn’t participate, we would never know, right? And if we didn’t have the research behind it, I think you would, there might be a higher reluctance… I can only speak to myself if a vaccine isn’t researched, I’m not having it. Do you know what I mean? It’s as simple as that. I’m not, I’m not having it. I’m not putting something unknown in my body without knowing potential side effects of that, or what it, whether it can do ‘what it says on the tin’ or all of that stuff. Do you know what I mean? So I do think research is important.”* Participant 1, Black Caribbean, 39 years old.

"*I think about population*, *so I think it’s important to*, *that these trials actually exist because the*, *the more individuals you can*, *um*, *involve in a study like this*, *closer you are to reality*.*”* Participant 18, South American, 32 years old.

While acknowledging the importance of participating in vaccine trials to demonstrate safety and effectiveness, participants described varying degrees of reluctance about their own involvement in such trials. Several participants described individuals who did participate in trials as ‘altruistic’ and expressed admiration for their contributions. However, when considering their own personal participation, most displayed some hesitation.

"*I know their importance, you know, it’s kind of, I have this, you know, like ethical dilemma that I’ll take, the good of the world depends on people doing these things, but I don’t want to do them. So like I understand there’s like a weird tension there that, when I’m saying out loud, it feels quite hard to resolve in my head. And so, yeah, I think my kind of going point is I would be hesitant on a normal day like pregnant or not and yeah.”* Participant 17, Mixed ethnicity (White and Asian), 34 years old.

All participants across the sample expressed their apprehensions about the potential adverse side effects on foetal development, a concern that transcended cultural boundaries. The majority of the participants from all backgrounds described how this anxiety, rooted in the maternal desire to protect their foetus, in combination with the increased perceived vulnerability of pregnancy acted as a de-motivator for participation in clinical trials.

"*I feel like when you’re pregnant taking part in a trial, it would be something I won’t put myself forward so much on. Whereas if I wasn’t pregnant, it’s just me, isn’t it? It’s just my body and I know my health and I know my health complications and history and stuff. Where with an unborn child I don’t know how that’s going to affect them, you know.”* Participant 10, Black African, 37 years old.

Furthermore, some participants were mindful of historic events, such as the thalidomide scandal, which left a lasting impression on their perceptions of the long-term safety of medical interventions carried out during pregnancy. The thalidomide scandal, occurring in the late 1950s and early 1960s, involved the widespread use of thalidomide to alleviate nausea in pregnant women, ultimately leading to severe birth defects in thousands of children [27]. We observed a heightened sense of responsibility for ensuring the long-term health and safety of their foetus, possibly informed by these previous events, shaping perceptions of the safety of medical interventions during pregnancy.

*"And you hear, like, will you scare stories about like, I didn’t know, like the thalidomide–I don’t even know how to say it- scandal or like things that they thought were fine at the time but then like 10 years later, you don’t know what’s going happen. And I’m more OK with taking that risk for just myself. But to take that for yourself and a child just seems like a bigger, a much bigger responsibility.”* Participant 5, South Asian, 33 years old.

Consequently, several participants emphasised the importance of receiving comprehensive information about the disease the vaccine being trialled was designed to prevent, as well as the safety data so far available to inform decision making about participation. While participants expressed a willingness to join trials in later stages once safety had been demonstrated, others were more inclined to adopt a cautious approach, awaiting further evidence of vaccine safety and regulation.

*"It depends on like the severity of the disease and like if it is like you know, if it can be without vaccine, if suppose, if I don’t take the vaccine and if that disease can be gone away like you know in one week or two week after taking the medicines, maybe I wouldn’t be. Or else like you know, depending on the complications, if you know if it has something like a serious complication or stuff like that I would be worried that I would not be taking part."* Participant 6, South Asian, 30 years old.

*"Like I’d I’m very happy to take vaccines*, *but once they have gone through trials and been approved*, *but I don’t know that kind of sounds a bit bad*, *but I also feel like I would be a bit sceptical to take part myself just because I wouldn’t*.*”* Participant 13, Mixed (White and Asian), 33 years old.

### Factors influencing participation in vaccine trials during pregnancy in those from ethnic minority backgrounds

#### Identity and belonging

Among the ethnically diverse participants interviewed, the intention to participate in vaccine trials was notably interlinked with their sense of identity and belonging. The concept of identity refers to self-perception and social recognition of an individual’s membership in a particular group or category. Belonging, on the other hand, denotes the feeling of acceptance, inclusion, and connection within a community [28]. For participants in the study, their participation in vaccine trials represented an act of solidarity with their communities, and a way to contribute to a collective responsibility for the public health of the community. Black pregnant women in particular described the impact of personal connection and community in shaping pregnant women’s decisions relating to vaccine trial participation in pregnancy.

*“I feel like Black people only react when it’s something that affects them specifically, they’ve had an experience of someone close to them and that’s when they go out their way to trial a vaccine…”* Participant 10, Black African, 37 years old.

*"It’s a tricky one*, *the only thing that probably comes to mind which think may well help is that …we have some illnesses that are more specific to certain ethnic minorities*. *For example*, *sickle cell*, *thalassaemia*, *examples like that*. *So if there was more talk around what affects your community more and this is why we would like you to take part in the vaccines because then your participation can*, *you know*, *inform our research and development of medication to help your community*. *Umm that may open people’s minds up a little bit more*.*”* Participant 21, Black Afro-Caribbean, 39 years old.

Furthermore, there was a suggestion that there were generational disparities influenced the sense of identity. Few participants, primarily second-generation migrants, explained that their parents who were born and raised in different countries, held distinct healthcare perceptions rooted in their unique identities and personal histories. In contrast, younger generations identified themselves as “western” and expressed more optimistic views on healthcare.

"*Um my parents are sort of first generation…they were born abroad and there wasn’t a very good health care system in Pakistan, for example, where we’re from. And so moving to a country where it’s Western doesn’t necessarily change your opinion about healthcare even though the system is completely different. So I think in their minds, even now when they attend doctor’s appointments and things, I often get phone calls saying, ‘oh the Doctor said this to me, I’m not sure how much I believe that, what’s your opinion about it’? Whereas I don’t have that experience of what healthcare system is like in Pakistan, so I don’t have a preconceived notion about, you know what that information is like. I would probably consider myself to be more Western than my parents and my siblings.”* Participant 3, South Asian, 30 years old.

Participants of Black and South-Asian backgrounds, emphasised the role that representation plays in guiding decision making for trial participation. They emphasised the importance of seeing individuals that they could identify with represented in healthcare settings and media campaigns. In the context of vaccine research, representation holds particular significance in addressing the historical underrepresentation of ethnic minority groups in clinical trials and healthcare institutions. This underrepresentation has fostered a perception among some Black and South-Asian participants that their concerns and needs are not being adequately considered by healthcare staff who may lack the cultural context or competency to alleviate the unique concerns they may have.

"*I really do think you need to have someone who looks like the your targeted audience, you know, giving your talks, your speeches or what have you and, and on top of that that, that person in short that they can actually relate to and understand kind of the challenges, the faults, or what have you, that those communities have. And you know, any fears they may have, and you can understand and provide that reassurance…”* Participant 1, Black Caribbean, 39 years old.

“*I think more involvement probably um in healthcare in general by people from minority groups*, *because then that increases the level of conversation that happens”* Participant 3, South Asian, 30 years old.

Participants’ responses emphasised the distinction between superficial and authentic representation that goes beyond tokenism. An example of superficial representation, cited by some participants was presenting ethnic minority individuals visually on research posters and leaflets. Meaningful representation was described as prioritising the involvement of ethnic minority members in research teams from the very inception of trials to ensure that their voices and concerns were included.

" *I think the people who are talking about the vaccine trials have to also have an understanding of like, what you’re doing now, have an understanding of how it the views that ethnic minorities have, and kind of then discussing and alleviating those fears and having those conversations. So a poster itself is not enough, but a conversation, a discussion goes a lot further, but obviously you have to get them involved first place."* Participant 21, Black Afro-Caribbean, 39 years old.

*“When you when you start the study it*, *it should be priority*. *We need to be represented*. *We need a seat on the table*.*”* Participant 10, Black African, 37 years old.

#### (Mis)Trust in healthcare

Participants’ views on healthcare systems shaped whether they would consider participating in vaccine research, particularly when pregnant. Trust and mistrust in health systems, shaped by past experiences of interacting with healthcare professionals informed whether pregnant individuals from ethnic minority communities were willing to consider participating in vaccine studies. Participants from Black and South Asian communities in particular were vocal about a lack of trust, leading people in their communities to question the motives and safety of these trials.

“*I think in my culture, there’s a lack of trust with healthcare professionals firstly. So any information that’s given from healthcare professionals is sort of questioned several times before it’s accepted.”* Participant 3, South Asian, 30 years old.

"*So there’s that distrust that I don’t know*, *since it’s been there*. *I don’t know how it can be solved*, *but it’s there*. *It’s feels like the vaccines or medications are targeted against*, *you know*, *other ethnicities*, *which is not true*, *but it’s there*, *it’s out there*.*”* Participant 22, Black African, 25 years old.

Mistrust appeared to be influenced by cultural backgrounds, as some participants expressed a high level of trust in healthcare professionals from their specific cultural perspective. These attitudes transcended cultural boundaries and remained intact even in different contexts.

“*Uh, in Brazil, there is this thing where they really trust a doctor, an MD. You had to be an MD. So when you say MD says somethings wrong and a nurse for example says somethings right, they are always going to deposit their trust in the end, in the MD, the doctor, the physician.”* Participant 18, South American, 32 years old.

Participants pointed out that attitudes to healthcare professionals may reflect broader attitudes towards governments and institutions. Healthcare services may be perceived indistinctly as a governmental institutions and therefore met with scepticism and mistrust.

"*And I think I find in any event the Black community are very, they’re very private individuals, not saying like anybody else isn’t. But when it comes to professionals, whether that be a doctor or whether that be any other kind of professionals there, I think you have to work harder at building up that trust within the Black community than you do, perhaps with another ethnicity, if I’m totally honest.”* Participant 1, Black Caribbean, 39 years old.

"*I don’t know*. *You know*, *I think there’s just like a more reluctance to be involved*, *with like authorities in general*. *So like any NHS is like a helpful thing*, *but a lot of people see it as like an interaction with a state authority*.*”* Participant 5, South Asian, 33 years old.

Participants also highlighted the need for healthcare professionals to work harder at relationship-building to gain trust. However, the task could be challenging due to the resource limitations in the healthcare system, with the majority of participants noting a lack of a continuous relationships with a midwife or GP.

“*Since I’ve moved to London, I don’t think I’ve had a relationship with any kind of medical person. I have a different GP every time, a different midwife and everything, a different person.”* Participant 4, South Asian, 33 years old.

This sentiment was echoed by several participants, with some suggesting that being approached about vaccine research by a familiar healthcare provider could encourage participation. An absence of continuity of care and poor outreach in hard-to-reach groups could exacerbate the issue of misinformation about vaccines. One participant highlighted the importance of researchers and healthcare workers actively reaching out to hard-to-reach communities, rather than expecting these communities to seek them out. They emphasised the significance of familiar healthcare providers engaging directly with communities in places with large populations of people from ethnic minorities.

*"But I feel like there needs to be like people coming into the community where there’s probably predominantly Black people, you know where we’re living, set up your van outside and spread the word in Lewisham Market or Brixton Market or Croydon and tell us why in more depth. And have our own people telling us, have Black people in those things, telling us this is why we need your blood; this is the type of stuff that you could save."* Participant 10, Black African, 37 years old.

Several participants also emphasised the impact of social media and the spread of misinformation on their trust in healthcare. One participant who had taken part in research when younger, viewed medical research more negatively now as a result of social media:

*“And I feel like again the reason why I took part in it (medical research) years ago is because social media wasn’t as big. Social media has taken over and everyone’s opinions on most things are just so negative, and it just threw me.”* Participant 12, Black Caribbean, 30 years old.

Additionally, some participants demonstrated a preference for holistic approaches, such as maintaining a natural diet and using herbal remedies. These individuals sought to leverage their cultural capital by adopting alternative health practices.

"*…with African culture, sometimes it’s more we need to do everything natural, herbal, things like that. So my partner would be like, well, if there’s a medication for it, there’s gonna be something natural for it we can use something herbal."* Participant 15, Black Caribbean, 31 years old.

#### Practical considerations

For those who expressed interest in trial participation, practical considerations such as excessive time commitments relating to study procedures, travel to and from study sites, and organising childcare, acted as a barrier to participation.

"*I guess it would also depend on how long the trial would be going on for and how much of my day-to-day and it would sort of take up. I’m generally work quite a lot so I’m sort of finding the time to actually access and sort of be on board with it. I wouldn’t want to sort of get involved in something and then not be able to commit to it properly.”* Participant 3, South Asian, 30 years old.

"*Umm*, *and then I would want to talk to my husband about it really just to say*, *if you know this thing I’m going to be doing takes time away or I need him to cover for the childcare or*.*”* Participant 2, South Asian, 36 years old.

The level of invasiveness associated with trial procedures held considerable importance as some participants expressed discomfort with invasive medical processes. Conversely, one participant identified that a potential incentive for their participation would be increased monitoring as part of a trial. This was seen as a countermeasure to the perceived increased personal risk that participants might be subject to as part of the trial.

*"One thing that would like entice me, I suppose, is the fact that you would get more, like if you could get like more monitoring, so in that way it feels like you get something out of it. If we feel that like to counteract that kind of feeling of it not being safe to be like, well, I get X amount more scans than you would have done.”* Participant 4, South Asian, 33 years old.

In this way, increased monitoring offered a tangible benefit that served to mitigate reservations about safety, increasing the appeal of participation in vaccine trials.

## Discussion

This study explored the barriers and enablers influencing the willingness of pregnant individuals from diverse ethnic backgrounds to participate in vaccine research studies during pregnancy. It is crucial to acknowledge that ethnic minority communities are highly diverse, as reflected in the study’s findings. Ethnicity encompasses multifaceted characteristics such as language, religious beliefs, and shared heritage, contributing to significant variations among ethnic groups [29].

Encouraging researchers to tailor their approaches in recruiting those from ethnic diverse communities for vaccine trials is vital. Applying recommendations with the aid of a community engagement facilitator familiar with relevant cultural contexts is crucial to foster trust. Despite the differences observed among different groups, the overarching themes discussed earlier should inform the development of vaccine research that is comprehensive and inclusive for those from ethnically diverse backgrounds.

The barriers identified in our study resonate with the existing literature on ethnic minority participation in clinical trials. Mistrust in healthcare professionals and research, particularly expressed by Black and South-Asian women, aligns with findings from previous studies in the UK [30–32]. Interestingly, our study suggests that pregnancy might not significantly affect these barriers.

This mistrust in healthcare settings, may have also impacted our ability to recruit to this study. Despite substantial recruitment efforts, the number of participants available for interviews fell short of our expectations. Our recruitment primarily took place within a hospital setting. As highlighted by one participant, healthcare workers and researchers should concentrate on reaching hard-to-reach communities in environments familiar to them. To enhance engagement with minority communities, recruitment strategies need to move away from hospitals, often perceived as untrusted spaces, and instead target areas where trusted sources are present. This is supported by a study of strategies used to improve engagement of ‘hard to reach’ groups of older adults in the UK [33]. This strategic shift could notably improve connections and research recruitment within these communities. Our recruitment strategy may not only have resulted in smaller number of participants being recruited, but potentially also leading to recruiting individuals who are more positive about vaccine trial involvement.

We found in our study the influence of misinformation, particularly spread through social media platforms, has contributed significantly to shaping perceptions and attitudes towards vaccines [34, 35]. Misinformation circulating on social media can potentially amplify the existing barrier of mistrust and further deter participation in vaccine trials during pregnancy. Addressing this challenge requires not only tailored approaches and active efforts to counter misinformation by disseminating accurate and accessible information through trusted community channels and reputable sources.

Additionally, it is vital that researchers reflect on how they are seen by pregnant people from different communities, for example whether they hold insider or outsider status [23], as well as considering the previous interactions which pregnant women may have experienced with health care professionals and systems. It should not be assumed that everyone has had positive experiences previously and researchers must not assume trust but rather design communication strategies which promote and build trusting relationships. Partnering with community experts and paying attention to relevant linguistic and cultural competencies is likely to enable research recruitment and participation [36, 37].

Finally, practical considerations cannot be underestimated when it comes to recruiting during pregnancy. Research shows that time constraints are perceived as a barrier to recruitment of individuals from ethnically diverse communities, and the use of incentives for an individuals’ time is likely to gain a higher rate of participation from minorities within research studies [38, 39].

This study has provided some valuable insights from under-researched populations of pregnant individuals from ethnic minorities. Strengths of the study include an ethnically diverse cohort that covered some of the most well known vaccine hesitant groups. We were also able to reach data saturation during the qualitative analysis process. There were also some limitations. We were unable to recruit anyone who was not able to communicate in English which means that we are unable to explore any of the attitudes of non-English speaking minorities. Our population was also probably more positive in general which reduced the generalisability of our findings. More research is necessary to build on our results, for example recruiting community champions and recruiting in community settings as well as in hospitals.

## Conclusion

Understanding the barriers and enablers influencing the willingness of pregnant individuals from ethnic minorities to participate in vaccine trials is crucial for developing inclusive and effective strategies that reduce inequalities in medical research and healthcare. Tailored interventions, focused on fostering trust, authentic representation, and addressing practical considerations, can contribute to enhancing vaccine trial participation among those from ethnic minorities, potentially reducing vaccine hesitancy and ultimately benefitting maternal and infant health outcomes.

## Supporting information

S1 File Consolidated criteria for reporting qualitative studies (COREQ) checklist. (DOCX)

S2 File Interview guide. (DOCX)
