# Supplementary material for: Understanding the barriers and enablers to participation in vaccine trials in a pregnant population from diverse ethnic background in an inner-city UK hospital
Source: PLoS One. 2024 Oct 30;19(10):e0312799. doi: 10.1371/journal.pone.0312799 (PMC11524452; doi:10.1371/journal.pone.0312799)
Supplement: S1 File — (DOCX) [file pone.0312799.s001.docx]

**S1 Table. Consolidated criteria for reporting qualitative studies (COREQ) checklist**

Developed from:

Tong A, Sainsbury P, Craig J. Consolidated criteria for reporting qualitative research (COREQ): a 32-item checklist for interviews and focus groups. Int J Qual Health Care. 2007;19(6):349-357.

| No. Item | Guide question/description | Reported on Page #* |
| --- | --- | --- |
| **Domain 1: Research team and reflexivity** |  |  |
| Personal characteristics |  |  |
| 1. Interviewer/ facilitator | Which author/s conducted the interview or focus group? | Page 6 |
| 1. Credentials | What were the researcher's credentials? E.g. PhD, MD | Page 6 |
| 1. Occupation | What was their occupation at the time of the study? | Page 6 |
| 1. Gender | Was the researcher male or female? | Page 6 |
| 1. Experience and training | What experience or training did the researcher have? | Page 6 |
| Relationship with participants |  |  |
| 1. Relationship established | Was a relationship established prior to study commencement? | Page 6 |
| 1. Participant knowledge of the interviewer | What did the participants know about the researcher? e.g. personal goals, reasons for doing the research | Page 7 |
| 1. Interviewer characteristics | What characteristics were reported about the interviewer/facilitator? e.g. Bias, assumptions, reasons and interests in the research topic | Page 7 |
| **Domain 2: study design** |  |  |
| Theoretical framework |  |  |
| 1. Methodological orientation and Theory | What methodological orientation was stated to underpin the study? e.g. grounded theory, discourse analysis, ethnography, phenomenology, content analysis | Page 5 |
| Participant selection |  |  |
| 1. Sampling | How were participants selected? e.g. purposive, convenience, consecutive, snowball | Page 6 |
| 1. Method of approach | How were participants approached? e.g. face-to-face, telephone, mail, email | Page 6 |
| 1. Sample size | How many participants were in the study? | Page 6 |
| 1. Non-participation | How many people refused to participate or dropped out? Reasons? | Page 6 |
| Setting |  |  |
| 1. Setting of data collection | Where was the data collected? e.g. home, clinic, workplace | Page 6, 7 |
| 1. Presence of non-participants | Was anyone else present besides the participants and researchers? | Page 7 |
| 1. Description of sample | What are the important characteristics of the sample? e.g. demographic data, date | Page 8 |
| Data collection |  |  |
| 1. Interview guide | Were questions, prompts, guides provided by the authors? Was it pilot tested? | Page 6, 7  S2 File Interview guide |
| 1. Repeat interviews | Were repeat interviews carried out? If yes, how many? | N/A |
| 1. Audio/visual recording | Did the research use audio or visual recording to collect the data? | Page 7 |
| 1. Field notes | Were field notes made during and/or after the interview or focus group? | Page 7 |
| 1. Duration | What was the duration of the interviews or focus group? | Page 7 |
| 1. Data saturation | Was data saturation discussed? | Page 6 |
| 1. Transcripts returned | Were transcripts returned to participants for comment and/or correction? | N/A |
| **Domain 3: analysis and findings** |  |  |
| Data analysis |  |  |
| 1. Number of data codes | How many data coders coded the data? | Page 7 |
| 1. Description of the coding tree | Did authors provide a description of the coding tree? | N/A |
| 1. Derivation of themes | Were themes identified in advance or derived from the data? | Page 7 |
| 1. Software | What software, if applicable, was used to manage the data? | Page 7 |
| 1. Participant checking | Did participants provide feedback on the findings? | N/A |
| Reporting |  |  |
| 1. Quotations presented | Were participant quotations presented to illustrate the themes / findings? Was each quotation identified? e.g. participant number | Page 8-17 |
| 1. Data and findings consistent | Was there consistency between the data presented and the findings? | Page 8-17 |
| 1. Clarity of major themes | Were major themes clearly presented in the findings? | Page 8-17 |
| 1. Clarity of minor themes | Is there a description of diverse cases or discussion of minor themes? | Page 8-17 |

*Page number corresponds to final manuscript submission pages.
