# Supplementary material for: Understanding the barriers and enablers to participation in vaccine trials in a pregnant population from diverse ethnic background in an inner-city UK hospital
Source: PLoS One. 2024 Oct 30;19(10):e0312799. doi: 10.1371/journal.pone.0312799 (PMC11524452; doi:10.1371/journal.pone.0312799)
Supplement: S2 File — (DOCX) [file pone.0312799.s002.docx]

**S2 File: Interview Guide**

Interview duration: 20- 30 minutes

Thank you for agreeing to speak to me. This interview will take around 20-30 minutes of your time. Please stop me to ask questions if anything is not clear.

The aim of this interview is to understand what your thoughts are about participating in vaccine research studies. There are no right or wrong answers and we are interested in your opinion.

With your permission, this interview will be audio recorded. All information you provide will be confidential, and any information that could identify you will be removed from all transcripts, and the audio will be deleted at the end of the study.

[*Note: allow prompting to clarify answers or to probe respondent’s thoughts further]*

Before we begin, do you have any questions or is anything not clear?

**Section 1: Perception and experiences of participating in vaccine research projects**

1. I would like to begin by asking your thoughts about participating in vaccine research.
2. Do you/would you usually consider participating in vaccine research projects?
3. Tell me more about why you would participate in vaccine research studies?
4. Tell me more about why you would choose NOT to participate in vaccine research studies?
5. What are your general reservations/hesitations towards participating in vaccine research?
6. Who do/would you discuss participation in vaccine research with? What do/would your loved ones (family and friends) say about participating in vaccine research?
   - Are they encouraging or apprehensive about you participating in vaccine research? Why?
   - Why do you think they think this way?
   - *(if brought up, prompt here about views from people from their community/ethnic group)*
7. Do you think vaccine research is important? Why do you say this?

**Section 2: Perception and experiences of participating in vaccine research projects while pregnant**

1. How likely would you be to take part in a vaccine research study while you are pregnant? Can you explain why?
2. What sort of studies would you consider participating in?
3. Is participating in vaccine research different when you are pregnant? Why? Why not?
4. What would your reservations be? And why?
5. If you feel this is a good idea, can you explain why?
6. Do you think vaccine research in pregnancy is important? Why do you say this?
7. What information would you need before making a decision to participate in vaccine research?
8. What do/would your loved ones (family and friends) say about participating in vaccine research while pregnant?
   - Are they encouraging or apprehensive about you participating in vaccine research? Why?
   - Why do you think they think this way?
   - *(if brought up, prompt here about views from people from their community/ethnic group)*
9. Do you think we can do anything different to encourage you to participate in trials while you are pregnant?
   - What would we need to do to encourage women to participate in research while they are pregnant?
   - What can we do? E.g., Give you more information? Make it easier to make decisions? Give more incentives?

**Finally, some questions about you:**

1. Have you had any children before? *(If no, move to question 20)*
2. How old are your children?
3. Have you participated in vaccine research before? During pregnancy?
4. Which ethnic group do you feel you belong to?
5. How long have you lived in the UK?
